# Optimized hidden target screening for very polar molecules in surface waters including a compound database inquiry

**DOI:** 10.1007/s00216-020-02743-0

**Published:** 2020-06-02

**Authors:** Susanne Minkus, Sylvia Grosse, Stefan Bieber, Sofia Veloutsou, Thomas Letzel

**Affiliations:** 1 Technical University of Munich (Chair of Urban Water Systems Engineering), Am Coulombwall 3, 85748 Garching, Germany; 2 Analytisches Forschungsinstitut für Non-Target Screening GmbH (AFIN-TS), Am Mittleren Moos 48, 86167 Augsburg, Germany; 3 Present Address: Thermo Fisher Scientific, Dornierstraße 4, 82110 Germering, Germany; 4 Present Address: N. Votsi 35, 10445 Athens, Greece

**Keywords:** Hydrophilic interaction liquid chromatography, High-resolution mass spectrometry, PMTs, vPvMs, DoE, Optimization

## Abstract

Highly polar trace organic compounds, which are persistent, mobile, and toxic (PMT) or are very persistent and very mobile (vPvM) in the aquatic environment, may pose a risk to surface water, ground water, and drinking water supplies. Despite the advances in liquid chromatography-mass spectrometry, there often exists an analytical blind spot when it comes to very polar chemicals. This study seeks to make a broad polarity range analytically accessible by means of serially coupling reversed-phase liquid chromatography (RPLC) and hydrophilic interaction liquid chromatography (HILIC) to high-resolution mass spectrometry (HRMS). Moreover, a workflow is presented using optimized data processing of nontarget screening (NTS) data and subsequently generating candidate lists for the identification of very polar molecules via an open-access NTS platform and implemented compound database. First, key input parameters and filters of the so-called feature extraction algorithms were identified, and numerical performance indicators were defined to systematically optimize the data processing method. Second, all features from the very polar HILIC elution window were uploaded to the STOFF-IDENT database as part of the FOR-IDENT open-access NTS platform, which contains additional physicochemical information, and the features matched with potential compounds by their accurate mass. The hit list was filtered for compounds with a negative log *D* value, indicating that they were (very) polar. For instance, 46 features were assigned to 64 candidate compounds originating from a set of 33 samples from the Isar river in Germany. Three PMT candidates (e.g., guanylurea, melamine, and 1,3-dimethylimidazolidin-2-one) were illustratively validated using the respective reference standards. In conclusion, these findings demonstrate that polarity-extended chromatography reproducibly retards and separates (very) polar compounds from surface waters. These findings further indicate that a transparent and robust data processing workflow for nontarget screening data is available for addressing new (very) polar substances in the aqueous environment.

**Electronic supplementary material:**

The online version of this article (10.1007/s00216-020-02743-0) contains supplementary material, which is available to authorized users.

## Introduction

Trace organic compounds (TOrCs) are widely known for being ubiquitous in the aquatic environment [1]. Recently, attention was drawn to one specific group of very polar and poorly degradable TOrCs—classified as “persistent in the environment, mobile in the aquatic environment, and toxic” (PMT) or “very persistent in the environment and very mobile in the aquatic environment” (vPvM)—because they give rise to environmental concerns [2, 3]. PMTs and vPvMs originate from household or industrial chemicals, pharmaceuticals, or pesticides, and enter the aquatic environment through point sources like municipal and industrial wastewater discharge [4] or diffuse sources such as runoff from agricultural fields or urban surfaces. Additionally, biotic or abiotic reactions from both metabolites and transformation products tend to be more polar than the precursor molecule [5]. Whereas nonpolar chemical compounds can often be efficiently eliminated by sorption to sludge or biotransformation/biodegradation, conventional wastewater treatment fails to significantly remove PMTs and vPvMs [6]. Being highly polar and thus mobile in water, these compounds spread along partially closed water cycles and are able to overcome technical and biological barriers. The result is that PMTs and vPvMs are high-risk candidates for eventually reaching drinking water supplies.

The advent of liquid chromatography (LC) coupled to mass spectrometry (MS) has created new opportunities for detecting polar substances in complex samples. Nevertheless, a large fraction of PMTs and vPvMs still constitute a blind spot in terms of analysis, monitoring, and regulation [7]. Recognition of this “analytical gap” led to an increasing demand for chromatographic techniques which complement classical reversed-phase liquid chromatography (RPLC). Supercritical fluid chromatography (SFC) appears to be a promising technology because it is able to separate compounds of a broad polarity range within a relatively small elution window [8]. Furthermore, hydrophilic interaction liquid chromatography (HILIC) seems to be predestined for coupled separations because of its orthogonality to RPLC [9, 10]. Alpert originally postulated a partitioning mechanism between a hydrated layer partially immobilized on a hydrophilic stationary phase and a relatively hydrophobic bulk eluent [11]. Later on, a theory of a multimodal separation was put forward in which retention occurs through partitioning interactions as well as hydrogen bonding and ion exchange [12, 13]. Despite having a complex retention mechanism, applications already exist which prove that HILIC can effectively separate very polar and environmentally relevant molecules [8, 14, 15]. In 2013, Greco et al. developed a serial coupling of RPLC and HILIC that takes advantage of the complementarity of both techniques and enables both highly polar and nonpolar compounds to be separated within a single run [16]. Injecting large volumes of aqueous sample on a HILIC column might render elution patterns. However, the injection on the RPLC column of the polarity-extended chromatographic system presented here overcomes this issue, and the final eluent containing a high acetonitrile content is very suitable for mass spectrometric electrospray ionization (ESI).

In order to widen the analytical window in favor of more polar molecules, the overall approach regarding how to handle a sample and the respective data requires adjustment. The nontarget screening strategy as initially introduced by Hérnandez et al. [17] and further refined by Krauss et al. [18] is well-suited for revealing unknown molecules of interest in complex matrices without prior information or reference standards. The German Water Chemical Society recently described it as a “[…] procedure without limitation to pre-select substances” [19]. The nontarget approach was essentially driven by the evolution of high-accuracy and high-resolution mass spectrometers (HRMS) able to acquire full scan data within a remarkably large mass range. However, even though high mass accuracy reduces the chance of erroneously assigning a molecular formula to a detected mass, further constraints need to be applied for an unambiguous allocation. Kind and Fiehn generated a comprehensive in silico test set of molecular formulae using the elements C, H, N, O, S, and P while complying with chemical and mathematical rules. They showed that the upper mass limit for unique formula assignment is still as low as 185.9760 Da at a theoretical accuracy of 0.1 parts per million (ppm) [20]. It becomes obvious that the results of a nontargeted measurement need to undergo further filtering steps. Criteria can be derived from instrumental boundary conditions and physicochemical properties specific to a compound group. In addition to the predefined mass range, chromatographic information like a retention time (RT) window and/or retention time indexing (RTI) also constitutes instrumental constraints beneficial to data filtering. In parallel with a suspect screening approach, the gathered data may be compared with entries of potential PMTs/vPvMs in a compound database. The search is facilitated by polarity indicators like the logarithmic distribution coefficient between octanol and water (log *D*). Substances which are known in chemical databases but unknown to the investigator are referred to as “known unknowns” [21] or “hidden targets” [22]. In contrast to the hidden target strategy, a suspect screening commonly targets the suspected ions already at the data acquisition stage, for instance, by conducting “multiple reaction monitoring” experiments.

Alongside the hidden target screening workflow—from planning an experiment to a final list of tentative PMTs/vPvMs found in a sample—several issues typically arise that require some form of compromise:

- Introducing statistical value to the conducted study versus maintaining feasibility in terms of total measurement time and HRMS data volume
- Reducing false positive allocations by applying a set of restrictive parameters and filters to the data processing method versus avoiding information loss by choosing overly rigid threshold values
- Optimizing the data processing method according to individual needs versus keeping the process transparent and reproducible

These conflicts pose a classical optimization problem and become apparent when composing a method for extracting features from nontarget screening data. Such a feature extraction (FE) method has several quantitative parameters and filters upon which the quality of the resulting feature list depends. Despite the complexity of setting these parameters, the common approaches include either using default settings or optimizing by “trial and error,” or changing “one factor at a time.” Not only do these approaches strongly rely on the investigator’s personal experience, they frequently also disregard statistical interactions between parameters. A more efficient and systematic approach is the statistical design of experiment (DoE): The maximum amount of information is extracted from complex nontarget screening data while simultaneously reducing experimental effort. The idea is to vary relevant peak picking parameters simultaneously over a reduced set of experiments. Cause-and-effect correlations are described by a mathematical model that can in turn be used for the interpretation, prediction, and optimization of these parameters. Since the pioneering work of Fisher in 1926 [23], the field has developed several methodological instruments such as the Plackett-Burman screening design [24], upon which the experimental plan used in this study was based.

In the following, an analytical workflow is suggested for facilitating the identification of PMTs and vPvMs in aqueous environments. Data was acquired by an RPLC-HILIC-ESI-time of flight (TOF)-MS coupling. Features were extracted by means of a systematically optimized method, then followed by a database inquiry and a filtering step for polar and very polar substances.

## Material and methods

In the following, polarity is approximated by the pH-dependent octanol-water partition coefficient log *D*. Substances are referred to as either “‘nonpolar’ for a log *D* > + 2, ‘polar’ for values between − 2.5 and + 2, or ‘very polar’ for a log *D* < − 2.5” at pH 7 as described elsewhere [8]. It should be kept in mind that a one-parameter coefficient like the log *D* can only describe the compound variability within a single substance class and does not consider interactions involved in partitioning [25].

### Chemicals

Acetonitrile and water (both LC-MS grade) were purchased from Fluka (Buchs, Switzerland). Reference compounds with a purity of > 99% were purchased from Sigma-Aldrich (Seelze, Germany). First, they were dissolved individually into stock solutions of 1 mM in either acetonitrile (nonpolar compounds) or in acetonitrile/water (50/50, v/v) (polar or very polar compounds). Afterwards, they were combined into a standard working mixture of 10 μM per compound.

### Water sampling and sample preparation

The samples used in this study were collected from the Isar river in Germany during March, May, and July 2015, respectively. Grab samples were taken at eleven locations between the Austrian-German border and the Bavarian city of Dingolfing, respectively (exact coordinates and descriptions of the sites are given in Table S1 in the Electronic Supplementary Material, ESM). Each monthly sampling pass and subsequent filtration were completed within a single day.

The samples were enriched using an offline polarity-extended solid-phase extraction (SPE) method. Therefore, two different extraction steps were sequentially conducted: (a) Reversed Phase (RP) Strata-X C18-endcapped from Phenomenex (Aschaffenburg, Germany) for nonpolar compounds and (b) ZIC-HILIC from Dichrom GmbH (Haltern am See, Germany) for (very) polar compounds. Firstly, the preconditioned RP cartridges were loaded with 150 mL of water sample. After passing through the column, it was freeze-dried with an Alpha 1 – 4 LSC freeze dryer (Christ, Osterode am Harz, Germany). The nonpolar fraction of the sample was eluted from the RP cartridge with 3 mL of acetonitrile/water (80/20, v/v), followed by 3 mL of pure acetonitrile. The eluate was dried in a vacuum until the solvents were completely evaporated. The remaining freeze-dried water sample was reconstituted in 12 mL acetonitrile/water (95/5, v/v) and centrifuged. Afterwards, the supernatant was loaded onto the preconditioned ZIC-HILIC cartridge. Three milliliters of acetonitrile/water (50/50, v/v) was used to elute the sample’s polar fraction from the ZIC-HILIC column into the tube containing the same dried nonpolar fraction of the sample. The combined extracts were dried again and finally reconstituted in 0.5 mL acetonitrile/water (50/50, v/v). The resulting solution was filtered through a 22 μM PVDF filter into a glass vial and stored at 4 °C prior to analysis.

Pure water (LC-MS grade) was processed the same way and served as a blank sample.

### Analytical instrumentation

The chromatographic separation was performed by a serial coupling of RPLC and HILIC. Detailed information on the setup of the system and the robustness thereof is provided elsewhere [15, 16].

In short, two binary pumps and two online degassers were used (Agilent Technologies, Waldbronn, Germany). The RP separation was performed on a Poroshell 120 EC-C18 (50.0 × 3.0 mm, 2.7 μm; Agilent Technologies) column and the HILIC separation on a ZIC-HILIC (150 × 2.1 mm, 5 μm, 200 Å; Merck Sequant, Umea, Sweden). The two columns were connected in series via a T-piece with a mixing frit (Upchurch, IDEX Europe GmbH, Erlangen, Germany). The third port of the T-piece was connected to a second binary pump. The following solvents were used as mobile phases: Solvent A was 10 mM ammonium acetate in water/acetonitrile (90/10, v/v) and solvent B was 10 mM ammonium acetate in acetonitrile/water (10/90, v/v) for the RPLC separation. For HILIC, acetonitrile (solvent C) and water (solvent D) were used. Information on the gradients is provided in Table S2 of the ESM. The injection volume was 10 μL.

The chromatographic system was coupled to an Agilent 6230 TOF-MS equipped with a Jet Stream ESI interface (Agilent Technologies, Santa Clara, CA, USA). The ESI source was operated in positive mode under the following conditions: gas temperature 325 °C, drying gas flow 8.0 L min^−1^, nebulizer gas pressure 45 psi, sheath gas temperature 250 °C, sheath gas flow 5.5 L min^−1^, capillary voltage 3 kV, fragmentor voltage 175 V. Nitrogen was used as both the drying and the sheath gas. During the analysis, a mass range up to 1700 m/z was scanned in high-resolution mode. The instrument was continuously calibrated on 125 nM purine and 6.25 nM HP-921 MS tuning mix (Agilent Technologies, Waldbronn, Germany) [16]. The instrument’s resolution is specified at 22,000 (full width at half maximum, FWHM) at m/z 1522 after automatic tuning procedure [26]. The accuracy was below 10 ppm.

Each sample was measured once. Interday repeatability of the chromatographic system was investigated on 68 standard substances by measuring them four times over the course of the campaign (ESM Table S3). For each compound, the mean value and standard deviations were calculated for RT, mass error, and the peak’s full width at half maximum (FWHM) and its height, respectively.

### Data processing and (tentative) molecule identification

The software that acquired the raw mass spectrometric data and controlled the system was MassHunter Workstation LC/MS Data Acquisition (version B.05.01, Agilent Technologies).

#### Feature extraction

Chromatographic peaks were binned together across all samples by RT and accurate mass. This compound bin is referred to as a feature which is characterized by the median values of RT, accurate mass, and signal intensity. Had MS/MS data been available, the spectrum would have been a further part of the feature.

The mass spectrometric raw data was processed by MassHunter Workstation Profinder (version B.06.00, Agilent Technologies) software. The feature extraction (FE) method is composed of two consecutive algorithms: The first which processes the HRMS data is an untargeted matter, deconvoluting all peaks that exceed an intensity limit defined by the investigator. Subsequently, ion species (molecular ions, isotopes, and adducts) that display the same chromatographic behavior are grouped together and aligned across all samples. Median values for masses, RTs, and composite spectra are calculated and fed into the second, recursive algorithm. This second algorithm uses these values to perform a targeted extraction and thereby improves reliability in features [27].

Within the above program, a “batch recursive FE” method for small molecules was built in order to generate a list of features. Each feature contains peaks of defined mass and RT aligned across the respective number of samples. Isotope grouping was performed on the basis of the common organic molecule model with a peak spacing tolerance of 0.0025 m/z + 7.0 ppm. The critical FE parameters thereby were optimized by means of DoE (see the “Optimization of the FE parameter using the design of experiment strategy” section), and the results are shown in Table 1.

**Table 1:** The robust setpoint is presented as a result of optimizing the critical FE parameters. For each quantitative factor, the respective value and the factor contribution were calculated

Quantitative factor	Value	Contribution (%)
F1	0 counts	9.0
F2	1	5.4
F3	0.87 min	46.1
F4	50 ppm	0.0
F5	0 counts	0.0
F6	5000 counts	2.0
F7	5000 counts	26.6
F8	10 ppm	10.9

#### Optimization of the FE parameter using the design of experiment strategy

**Fig. 1:**
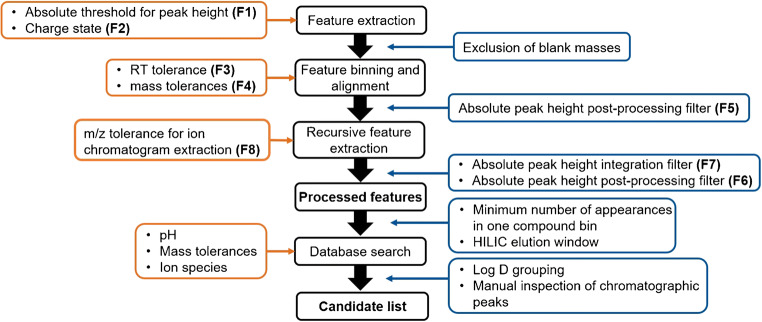
Workflow to evaluate nontarget screening data including a compound database search. Input parameters and filtering steps which significantly influence the output after each step are depicted in orange and blue boxes, respectively. The bolt numbers in brackets mark the critical parameters that were optimized

Prior to the FE, critical method parameters referred to as quantitative factors (F) were identified and optimized by means of DoE using MODDE Pro software (version 12.1.0.5491; Sartorius Stedim Biotech GmbH, Göttingen, Germany). The optimization was based on the HRMS raw data from a subset of six river water samples (due to extensive data processing times): The March and July samples were each taken at locations 1, 7, and 10 (ESM Table S1), respectively. The following critical quantitative FE factors were optimized and are illustrated in Fig. 1:

1. Absolute threshold for peak height prior to the FE
2. Charge state of the ions
3. RT tolerance for the binning and alignment of features
4. Mass tolerance for the binning and alignment of features
5. Absolute peak height filter applied after the untargeted algorithm
6. Absolute peak height filter applied after the targeted algorithm
7. Absolute peak height filter after integration
8. Symmetric m/z tolerance for the recursive chromatogram extraction

These eight FE factors were varied according to a strategically designed experimental plan of 28 runs which is provided in Table S4 of the ESM. Optimization was based on six response factors (R) in the form of numerical descriptors which were chosen to assess the performance of the FE method:

1. The RT span of all peaks binned into a compound group across all samples. After every experimental run, the median value was calculated for the final feature list
2. The relative standard deviation (RSD) of masses within a compound group across all samples. Again, the median value was calculated
3. The number of single ions relative to the total number of extracted features
4. The mass difference between the median mass within a compound group and the target mass used by the recursive algorithm
5. The number of erroneously integrated chromatographic peaks out of a sample of ten features, which covered broad mass and RT ranges and appeared in more than ten experiments (ESM Table S5). The extracted ion chromatograms (EICs) were checked manually
6. The number of missed chromatographic peaks out of the same sample of ten features

For each experiment, the response variables were calculated from the extracted feature list out of the sample subset. A model was fitted using multiple linear regression. The optimization was based on desirability functions with the objective of minimizing all responses. These desirability functions ran on the specification limits for each response variable given in Table S6 (see ESM) along with the experimental results. To search for the robust setpoint, a design space was generated with a resolution of 8, with 1000 iterations and a 1% acceptance limit.

#### (Tentative) molecule identification

**Fig. 2:**
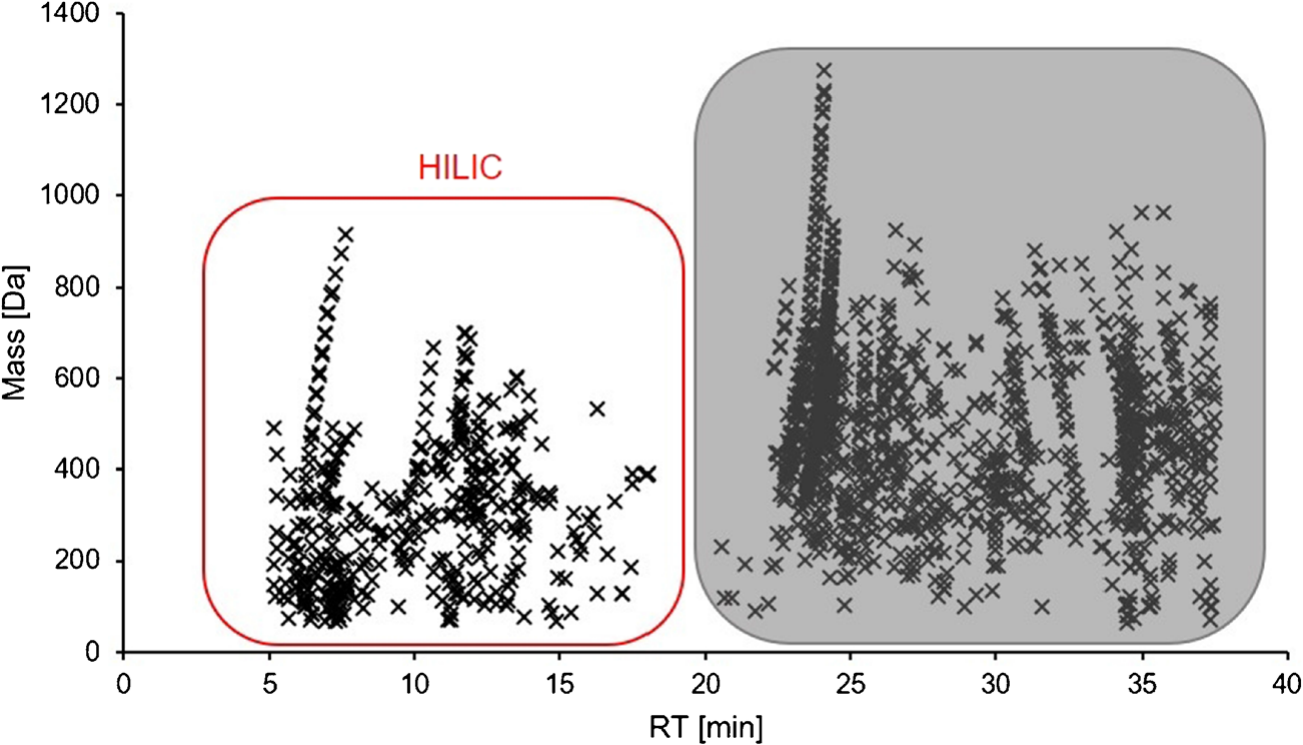
Mass-RT plot of features obtained from measuring 33 environmental samples with a RPLC-HILIC-TOF-MS coupling. All blank masses were excluded in the extraction method. The features can be separated into two distinct groups, the first of which represents those that eluted from the HILIC column

The exact masses of the features retained by the HILIC column were uploaded and processed by the FOR-IDENT platform [28] using the STOFF-IDENT compound database [29]. The transition from the HILIC to the RPLC retention window was between 16 and 21 min (Fig. 2). A hard limit was defined at the RT of metformin 17.0 min since it was the last standard compound eluting from the HILIC column (ESM Table S3). A pH level of 7 and a 10.0 ppm mass tolerance for the molecular ion were selected as input parameters. The resulting list of compounds, including their physiochemical properties, was downloaded. That list was then filtered for compounds with a log *D* value (at pH 7) below 0. Afterwards, the underlying chromatographic peaks of successfully matched features were manually checked. Compound lists were processed in Microsoft Excel (version 1902).

## Results and discussion

This study sought to combine a polarity-extended chromatographic method, i.e., the serial coupling of RPLC and HILIC, using a hidden target screening approach for tentatively identifying PMTs/PvMs.

A set of environmental data was used in order to exemplify a novel data processing and molecule identification strategy for very polar anthropogenic compounds. It is being presented with an emphasis on key parameters for ensuring transparency and reproducibility. Consequently, the study focused on data processing and tried to explicitly highlight the importance of transparently communicating the FE method. Since a large variety of data analysis software tools exist, the key peak picking parameters need to be identified and clearly stated. Therefore, a data-specific optimization is suggested for parameters and filters which are critical to extracting features from HRMS raw data. Subsequently, a compound database was consulted in order to tentatively identify (very) polar molecules by comparing physiochemical properties of the observed analytical data with compounds stored in the database. The data processing strategy for going from mass spectrometric raw data to a list of potential compounds of the very polar fraction of the sample is illustrated in Fig. 1. Every step along the workflow is characterized by several input parameters (orange boxes) and filters (blue boxes) acting as variables influencing the outcome in the form of a feature list.

Features are characterized by the median RT, accurate mass, and signal intensity, as described in the experimental section.

### Environmental samples studied

The workflow was built on 33 environmental samples that were taken during early 2015 from the Isar river in southern Germany (Bavaria). The eleven sites sampled started at the German-Austrian border close to the river’s source, then covered the urban area of Munich and finished in the town of Dingolfing. The exact locations are specified and commented on in Table S1 (see ESM). Each sampling pass was performed during a single day and repeated during the months of March, May, and July, respectively. Karakurt et al. [30] calculated the relative wastewater effluent contribution at different gauging stations along the Isar river from the accumulated discharge of upstream wastewater treatment plants under mean minimum annual discharge conditions. Their results showed that the wastewater effluent contribution was < 1% in Mittenwald, near the source. It jumped from 5 to > 50% after the plants treating the wastewater of Munich and recovered to 11% at the gauging station of the town of Landshut, 40 km downstream (probably due to dilution) [30]. Judging from the substantial portions of treated wastewater in densely populated areas, an elevated amount of anthropogenic PMTs and vPvMs was expected in the samples taken shortly downstream of Munich. Furthermore, this campaign was designed to unravel seasonal differences between the spring and summer samples.

The hidden target screening approach is well-suited for such global surveys of surface waters because it creates options for comparing samples by their molecular fingerprint. The aqueous samples were analyzed using the polarity-extended chromatographic separation technique RPLC-HILIC coupled with accurate TOF-MS.

### Analytical robustness

Robust operation of the analytical setup is a prerequisite for successfully handling the observed nontarget screening data and the evaluation thereof.

As a result, the data on the interday repeatability of the RPLC-HILIC-TOF coupling is presented in Table S3 of the ESM.

For the 43 standard compounds eluting from the RPLC column between 23.7 and 35.1 min, the mean standard deviation of RT was 0.3 min (RSD 0.9%). Norfluoxetine showed the largest variability at 0.6 min (2.1%). The mass spectrometric accuracy can be expressed by the mass error which corresponds to the relative deviation of the detected mass from the monoisotopic mass. All RPLC standard compounds displayed a mean mass error of < 7 ppm.

Twenty-five compounds were retained on the HILIC column and eluted between 5.9 and 17.0 min. The mean standard deviation of RT was 0.2 min (1.9%) whereas gabapentin and metformin exhibited the highest values at 0.5 min (4.6%) and 0.8 min (4.4%), respectively. The mean mass error was below 9 ppm for all HILIC compounds, which is in good agreement with an expected accuracy of 10 ppm for the applied time of flight mass spectrometer.

The overall scattering of the RTs for the standard compounds is considerably low, which indicates a reliable and robust separation of both the RPLC and the HILIC parts. Retention time stability is an important parameter when dealing with nontarget screening data since it directly factors into the componentization during the feature-finding process. Accordingly, for all features detected in the real samples during this 5-month project, the RSD of RTs was below 4% (Table 2). Moreover, the mass errors observed in the test set justify adopting a maximum mass difference of 10 ppm as a criterion for matching features with database compounds by accurate mass. More details on stability testing of the coupling can be found in the previous work published by Bieber et al. [8].

**Table 2:** Features described by their detected accurate masses and mean RTs with RSDs. They are presented along with the respective database hit(s). The features eluted from the HILIC column of the RPLC-HILIC-TOF-MS coupling and therefor only compounds with a negative log *D* value were considered

ID	Detected median mass	Detected mean RT (min)	Number of samples	RSD RT (%)	Compound name (STOFF-IDENT)	InChi key	Relative mass difference (ppm)	Log *D* (pH 7)
1	75.0688	13.8	10	0.3	3-Aminopropan-1-ol	WUGQZFFCHPXWKQ-UHFFFAOYSA-N	5.3	− 3.9
					1-Aminopropan-2-ol	HXKKHQJGJAFBHI-UHFFFAOYSA-N	5.3	− 3.4
					2-Methylaminoethanol	OPKOKAMJFNKNAS-UHFFFAOYSA-N	5.3	− 3.6
					2-Amino-1-propanol	BKMMTJMQCTUHRP-UHFFFAOYSA-N	5.3	− 3.5
2	102.0547	14.6	13	1.6	Guanylurea	SQSPRWMERUQXNE-UHFFFAOYSA-N	4.9	− 2.1
3	103.1001	12.4	3	0.6	3-Ethoxypropylamine	SOYBEXQHNURCGE-UHFFFAOYSA-N	3.9	− 3.0
					1-Dimethylaminopropan-2-ol	NCXUNZWLEYGQAH-UHFFFAOYSA-N	3.9	− 2.3
					3-Dimethylaminopropan-1-ol	PYSGFFTXMUWEOT-UHFFFAOYSA-N	3.9	− 2.6
4	109.0532	7.4	9	0.2	(1R,4S)-2-Azabicyclo[2.2.1]hept-5-en-3-one	DDUFYKNOXPZZIW-UHFFFAOYSA-N	3.7	− 0.2
					Nicotinylalcohol	MVQVNTPHUGQQHK-UHFFFAOYSA-N	3.7	0.0
5	113.1205	11.0	3	2.8	1-Ethylpiperidine	HTLZVHNRZJPSMI-UHFFFAOYSA-N	0.9	− 1.5
6	114.0795	5.6	11	0.5	1,3-Dimethylimidazolidin-2-one	CYSGHNMQYZDMIA-UHFFFAOYSA-N	1.8	− 0.6
7	115.0627	12.1	11	0.8	N-(Hydroxymethyl)methacrylamide	DNTMQTKDNSEIFO-UHFFFAOYSA-N	− 5.2	− 0.2
					4-Morpholinecarbaldehyde	LCEDQNDDFOCWGG-UHFFFAOYSA-N	− 5.2	− 0.9
					N-(2-Hydroxyethyl)prop-2-enamide	UUORTJUPDJJXST-UHFFFAOYSA-N	− 5.2	− 0.7
					Methyl-3-aminocrotonate	XKORCTIIRYKLLG-ONEGZZNKSA-N	− 5.2	− 0.24
8	115.0995	7.9	15	1.7	2,6-Dimethylmorpholine	HNVIQLPOGUDBSU-UHFFFAOYSA-N	− 1.7	− 1.3
					Trans-4-Aminocyclohexan-1-ol	IMLXLGZJLAOKJN-UHFFFAOYSA-N	− 1.7	− 3.0
					2-Pyrrolidin-1-ylethanol	XBRDBODLCHKXHI-UHFFFAOYSA-N	− 1.7	− 2.7
9	117.0798	11.7	30	0.8	l-Valine	KZSNJWFQEVHDMF-BYPYZUCNSA-N	6.8	− 2.0
					l-Norvaline	SNDPXSYFESPGGJ-BYPYZUCNSA-N	6.8	− 1.9
					2-Hydroxy-N,N-dimethyl-propanamide	YEBLAXBYYVCOLT-UHFFFAOYSA-N	6.8	− 0.8
10	121.0883	5.2	8	0.1	d-alpha-Methylbenzylamine	RQEUFEKYXDPUSK-SSDOTTSWSA-N	− 6.6	− 1.0
					Benzyl(methyl)amine	RIWRFSMVIUAEBX-UHFFFAOYSA-N	− 6.6	− 1.1
					l-alpha-Methylbenzylamine	RQEUFEKYXDPUSK-ZETCQYMHSA-N	− 6.6	− 1.0
					Phenethylamine (PEA)	BHHGXPLMPWCGHP-UHFFFAOYSA-N	− 6.6	− 1.2
11	126.0651	7.2	5	1.6	Melamine	JDSHMPZPIAZGSV-UHFFFAOYSA-N	− 2.4	− 2.0
	126.0655	6.9	4	0.5			0.8	
	126.0665	8.4	9	0.2			8.7	
12	129.0182	6.3	8	2.9	1,3,5-Triazinane-2,4,6-trione	ZFSLODLOARCGLH-UHFFFAOYSA-N	6.2	− 1.1
13	135.0554	7.7	4	0.3	Adenine	GFFGJBXGBJISGV-UHFFFAOYSA-N	6.7	− 0.6
14	144.0904	6.3	17	2.1	N,N′-Ethylenedi(diacetamide)	WNYIBZHOMJZDKN-UHFFFAOYSA-N	3.5	− 1.8
15	149.0709	7.6	4	0.6	3-Methyladenine	ZPBYVFQJHWLTFB-UHFFFAOYSA-N	5.4	− 0.3
					1-Methyladenine	SATCOUWSAZBIJO-UHFFFAOYSA-N	5.4	− 0.5
					9-Methyladenine	WRXCXOUDSPTXNX-UHFFFAOYSA-N	5.4	− 0.3
					7-Methyladenine	HCGHYQLFMPXSDU-UHFFFAOYSA-N	5.4	− 0.4
16	149.1046	12.2	10	1.0	2,2′,2″-Nitrilotriethanol	GSEJCLTVZPLZKY-UHFFFAOYSA-N	− 4.0	− 3.3
					2-(Dimethylamino)-2-(hydroxymethyl)propane-1,3-diol	FGLZHYIVVZTBQJ-UHFFFAOYSA-N	− 4.0	− 3.4
17	149.1052	11.2	17	1.0	2,2′,2″-Nitrilotriethanol	GSEJCLTVZPLZKY-UHFFFAOYSA-N	0.0	− 3.3
					2-(Dimethylamino)-2-(hydroxymethyl)propane-1,3-diol	FGLZHYIVVZTBQJ-UHFFFAOYSA-N	0.0	− 3.4
18	155.1311	8.6	19	3.0	2,2,6,6-Tetramethyl-4-piperidone	JWUXJYZVKZKLTJ-UHFFFAOYSA-N	0.6	− 0.3
					2-[(Dimethylamino)methyl]cyclohexanone	QDHLEFBSGUGHCL-UHFFFAOYSA-N	0.6	− 0.3
19	155.1316	6.2	17	1.7	2,2,6,6-Tetramethyl-4-piperidone	JWUXJYZVKZKLTJ-UHFFFAOYSA-N	3.9	− 0.3
					2-[(Dimethylamino)methyl]cyclohexanone	QDHLEFBSGUGHCL-UHFFFAOYSA-N	3.9	− 0.3
20	161.1413	15.1	3	0.9	2-[2-(Diethylamino)ethoxy]ethanol	VKBVRNHODPFVHK-UHFFFAOYSA-N	− 1.9	− 2.2
					(2,2-Diethoxyethyl)dimethylamine	SSFAUOAQOOISRQ-UHFFFAOYSA-N	− 1.9	− 0.1
					7,7-Dimethyl-3-oxa-6-azaoctan-1-ol	YDEDDFNFQOPRQJ-UHFFFAOYSA-N	− 1.9	− 2.6
21	163.0859	15.0	6	0.5	Bicine	FSVCELGFZIQNCK-UHFFFAOYSA-N	8.6	− 4.4
					3,4,5-Piperidinetriol, 2-(hydroxymethyl)-, (2R,3R,4R,5S)-	LXBIFEVIBLOUGU-JGWLITMVSA-N	8.6	− 4.0
22	170.0932	7.3	3	0.4	(1R,3S)-2,2-Dimethyl-3-(2-oxopropyl)cyclopropanecarboxylic acid	BKUHNVYPRVILOX-BQBZGAKWSA-N	− 6.5	− 1.6
23	171.0907	7.9	8	0.3	Ethyl 2-oxopyrrolidine-1-acetate	AQZWKPDVWWJWRY-UHFFFAOYSA-N	7.0	− 0.4
24	187.1211	8.1	10	0.2	(3R)-3-(2-Amino-2-oxoethyl)-5-methylhexanoic acid	NPDKTSLVWGFPQG-UHFFFAOYSA-N	1.6	− 1.5
25	191.1518	6.6	11	1.8	1,1′,1″-Nitrilotripropan-2-ol	SLINHMUFWFWBMU-UHFFFAOYSA-N	− 1.6	− 2.9
	191.1523	8.3	3	1.3			1.1	
26	201.1733	9.5	33	2.1	4-Hydroxy-2,2,6,6-tetramethylpiperidine-1-ethanol	STEYNUVPFMIUOY-UHFFFAOYSA-N	2.0	− 2.6
	201.1734	10.9	11	0.3			2.5	
	201.1737	8.4	3	2.1			4.0	
	201.1740	9.8	5	0.7			5.5	
	201.1742	10.9	11	0.3			6.5	
27	205.0599	11.8	6	0.9	Methylglycinediacetic acid	CIEZZGWIJBXOTE-UHFFFAOYSA-N	6.3	− 9.9
28	205.1304	7.7	7	0.5	Panthenol, dl-form	SNPLKNRPJHDVJA-UHFFFAOYSA-N	− 4.9	− 1.7
					Dexpanthenol	SNPLKNRPJHDVJA-ZETCQYMHSA-N	− 4.9	− 1.7
29	205.1327	5.7	11	0.1	Panthenol, dl-form	SNPLKNRPJHDVJA-UHFFFAOYSA-N	6.3	− 1.7
					Phenformin	ICFJFFQQTFMIBG-UHFFFAOYSA-N	0.0	− 3.8
					Dexpanthenol	SNPLKNRPJHDVJA-ZETCQYMHSA-N	6.3	− 1.7
30	221.1402	12.2	5	0.2	3,4-Methylenedioxypropylamphetamine	LBXMQBTXOLBCCA-UHFFFAOYSA-N	− 6.3	− 0.3
31	222.1475	6.0	7	0.3	Bis(2-(2-Methoxyethoxy)ethyl) ether	ZUHZGEOKBKGPSW-UHFFFAOYSA-N	3.6	− 0.1
					2-Ethyl-1-(2-(1,3-dioxanyl)ethyl)-pyridinium	HNGQYAWKWBGGDR-UHFFFAOYSA-N	− 6.3	− 2.4
32	234.1106	6.6	8	3.9	2,2′-[Ethane-1,2-diylbis(oxy)]bisethyl diacetate	OVOUKWFJRHALDD-UHFFFAOYSA-N	1.3	− 0.4
33	257.1052	13.7	9	0.7	Tolmetin	UPSPUYADGBWSHF-UHFFFAOYSA-N	0.0	− 0.2
34	267.1475	12.5	8	0.6	Metoprolol acid	PUQIRTNPJRFRCZ-UHFFFAOYSA-N	1.5	− 1.2
35	283.1775	12.0	11	0.1	Alpha-Hydroxymetoprolol	OFRYBPCSEMMZHR-UHFFFAOYSA-N	− 3.2	− 1.7
36	360.2144	12.1	7	0.3	Butylscopolamin	YBCNXCRZPWQOBR-FAQYLHNASA-N	− 7.0	− 1.9
					Butylscopolammonium	YBCNXCRZPWQOBR-MWGADRMYSA-N	− 7.0	− 1.9
37	362.1668	9.9	16	0.5	Protirelin	XNSAINXGIQZQOO-SRVKXCTJSA-N	− 9.7	− 3.4
38	468.2676	11.6	4	0.2	6,6′,6″-(1,3,5-Triazine-2,4,6-triyltrimino)tris-hexanoic acid	BKKWPPMEXIXECW-UHFFFAOYSA-N	− 4.3	− 5.3
39	475.2971	6.4	22	2.2	Netilimicin	CIDUJQMULVCIBT-IULVMANBSA-N	− 7.4	− 12.8

### Data processing strategy and optimization

#### Optimization of FE parameters and filters

The two FE algorithms applied have differing objectives:

- The nontargeted one that should allow a less restricted search in order to reduce the number of missed chromatographic peaks and
- the targeted one that should be more precise in order to introduce statistical confidence to the features. Since the target criteria of the second algorithm depend on the componentization of the first one, the parameter settings need to be coordinated.

Therefore, critical input parameters and filters (called factors) were identified and optimized before applying the FE method to real samples. The optimization was based on six responses which characterize the performance of a certain combination of factors: R1, R2, and R3 (see the “Optimization of the FE parameter using the design of experiment strategy” section for a definition) assess the quality of the feature componentization. Consequently, given closer binning and alignment tolerances, the percentage of single ions (R3) is likely to increase. Nevertheless, a fraction of observed single ions might also derive from a low signal intensity. R4 provides information on the accuracy of the recursive algorithm. R5 and R6 indicate whether integrated peaks are correctly allocated to a compound bin.

Based on the results of all 28 experiments included in the strategically designed plan, a model was fitted for each individual response by means of multiple linear regression. For model diagnostics, the coefficient of determination *R*^2^ was considered. The latter represents the fraction of a response that can be explained by the model. The predictive ability of a model can be estimated by *Q*^2^ [31]. For each response, *R*^2^ was > 0.7 and *Q*^2^ > 0.6. Furthermore, the FE algorithms generate the same feature lists for repeated processing attempts given that the same data set was used, and the same parameter values were set. Considering all three performance characteristics (*R*^2^, *Q*^2^, and reproducibility), the model was considered to be sufficiently significant. The formula for *R*^2^ and *Q*^2^, along with the individual values for each response, can be found in Table S7 (see ESM). Moreover, the model terms are also listed therein along with their respective coefficients.

A robust setpoint for the critical FE input parameters and filters was iteratively calculated with the objective of minimizing the six response variables. The resulting factor settings are presented in Table 1. Some results can be transferred to other data analysis workflows applied for HRMS, e.g., setting the initial threshold for the signal intensity (F1) to zero in order to decrease the number of missed chromatographic peaks while simultaneously applying a rigid post-processing filter (F6, F7) in order to reduce the number of falsely integrated peaks and single ions. Further findings are specific to the data set at hand, the measurement campaign which was conducted over a period of 5 months. The chromatographic and mass spectrometric conditions shifted slightly as a result. It is generally accepted that tolerance windows need to be implemented in feature-building algorithms which account for these RT and mass drifts [32]. Accordingly, an optimized EIC tolerance (F8) of 10 ppm was calculated from the model and is in good agreement with the mass errors found for the HILIC standard compounds in the TOF system (see “Environmental samples studied” section and ESM Table S3). Even though the average interday RT variability over the 25 standard compounds was < 2%, in some rare cases (such as for gabapentin and metformin), the RT shifted over an absolute span of > 1.0 min. As a result, an optimal and robust binning and alignment tolerance of 0.9 min was determined for RT (F3). Another way of coping with shifting instrumental conditions would be to split data sets into homogeneous blocks, with each block containing data from successively measured samples. On the one hand, this approach could lead to smaller tolerance windows for RT and accurate mass. On the other hand, however, it would mean an inability to handle the data from a consecutive campaign as one batch.

These findings prove that FE parameter settings need to adapt to fluctuating data quality. In this context, the concept of optimizing these parameters by DoE introduces flexibility into a workflow that processes nontarget data. A DoE approach is inherently accompanied by a thorough statistical evaluation of a parameter’s significance, effect, or potential interactions with other parameters and, as a result, leads to a better understanding of the algorithm in general. Examples of DoE are already being used for the purpose of optimizing peak picking parameters for XCMS software [33] in metabolomics [34, 35]. The present study shows that the concept is not limited to specific software and could serve as an add-on for improving a preexisting nontarget or hidden target screening strategy in environmental analysis. Moreover, it has the potential to become a fully automated element within an FE workflow.

#### Polarity-dependent feature extraction

The final FE method was applied twice: First, on the raw HRMS data of the blank sample and afterwards to the real sample set. The masses found in the blank sample were excluded from the feature list of the real river water samples. That way, 179 features were eliminated from the sample results table. One would anticipate more lost features after a blank exclusion compared with a blank subtraction, but there actually appears to be no significant differences between both methods [36]. One other option would be to define a sample-to-blank intensity ratio. However, blank exclusion is easier to implement into automated workflows. As depicted in the RT-mass plot in Fig. 2, the remaining 1739 features can be separated into two groups: The first group represents compounds retained by the HILIC column and the second those retained by the RPLC column. Thus, the features eluting in the first section of the run were expected to be polar or very polar [8]. Accordingly, all features with RTs > 17.0 min were eliminated from the feature list. Preliminary tests showed that the last very polar reference compound eluted from the HILIC column at that time (ESM Table S3).

The RPLC fraction of features that were detected by the serial coupling was not further considered in this study. Consequently, the instrumental setup implicitly set boundary conditions that could be used as a filter criterion with respect to the research question at hand.

Features able to be found in fewer than three different water samples were excluded in a further filter step prior to the database search. Although triplicates are in contemporary nontarget analysis considered to be the optimal with regard to replicate injections [36], each sample was measured only once. An elevated number of samples in addition to the already high data output of a nontargeted HRMS screening requires measures for increasing the feasibility of studies while simultaneously minimizing the number of false positive allocations. In the aforementioned study from 2015, the statistical need for triplicate injections of the same sample was not clearly proven and, further, single measurements reduce the expenditure of measurement time and resources. Instead, a threshold for features was introduced specifying the minimum number of appearances across all samples. The approach of filtering by “detection frequency” in real samples has been successfully applied in order to prioritize features for further investigations under different research questions [37, 38]. It works well for large sample sets, as was the case in this study, because eleven locations were sampled at several dates over 1 year (see ESM Table S1). In addition to the binning and alignment of peaks within a sample group, such a detection frequency threshold introduces more statistical confidence to a feature. However, this approach is not applicable in all situations since, for example, short-term emission of TOrCs from point sources will be missed. The number of eligible features was reduced to 408 after removing those eluting later than 17.0 min and those that were detected in fewer than three samples.

### (Tentative) compound identification in water samples

#### Inquiry of a compound database

These features were uploaded to the open-access platform FOR-IDENT handling the compound database STOFF-IDENT. Providing physicochemical properties of more than 11,000 anthropogenic compounds, this database was developed to support identification of pollutants and emerging contaminants relevant to the aquatic environment [39]. The database search was performed based on accurate mass ± 10 ppm on the platform. Thus, 132 features were successfully located in the database, and 287 anthropogenic trace organic compounds suggested. The difference derives from the given mass window, so multiple compounds were matched with a single feature. In some cases, it was the other way around: Various features were assigned to one compound. A list of compounds was proposed together with information on their polarity expressed by pH-dependent log *D* value. The list served as a basis for further filtering and investigation of the underlying peaks.

#### Polarity filtering of the FOR-IDENT results

Due to the complex retention mechanism of HILIC, the relationship between the analytes’ RTs and the related log *D* values has yet to be determined in detail. However, Bieber et al. tested a mix of 262 standard compounds on the RPLC-HILIC coupling and found that all of the 136 compounds that eluted from the HILIC column have a negative log *D* value [8]. In the current investigation, this was also true for the 25 reference compounds that eluted earlier than 17.0 min (ESM Table S3). Knowing that, 287 database hits were filtered for compounds that have a log *D* value < 0 at a pH value 7. One hundred nine remaining compound candidates had polar to very polar properties as indicated by their negative log *D* values and consequently were expected to elute from the HILIC column.

#### Manual evaluation

Finally, the 68 features that led to the 109 candidates were manually reevaluated in order to ensure that they originated from chromatographically acceptable peaks. For a feature to be classified as “acceptable,” it had to fulfill the following criteria:

- The spread of relative mass differences within the compound bin of the feature is lower than ± 10 ppm.
- The chromatographic peaks are approximately Gaussian shaped.
- Each peak’s mass spectrum comprises at least two ions to increase the reliability of single features.
- For at least one chromatographic peak in the compound bin, the peak’s mass spectrum displays an isotopic pattern.

Our approach assigned 64 PMT/vPvM candidates to 46 features (Table 2) detected in Isar river samples from March, May, and July of 2015. The numerous filtering steps for feature lists throughout this workflow reduced the effort of manually evaluating chromatographic peaks. Nevertheless, 18 features had multiple components allocated to them. That number could probably have been reduced by comparing MS/MS spectra. A validation with reference standards is still pending at this time.

**Fig. 3:**
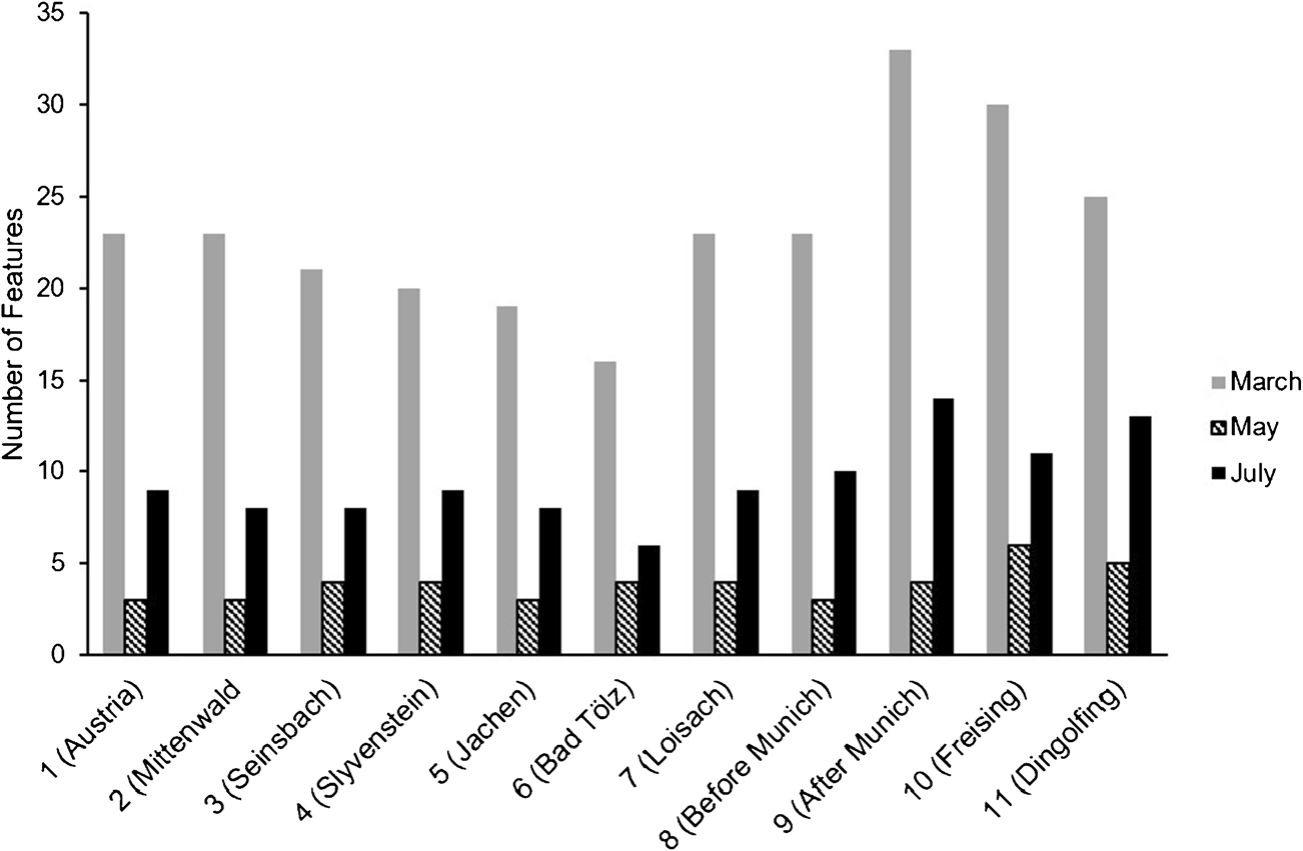
Spatial distribution along the Isar river of 46 features found in 33 environmental samples of 2015. They represent the portion of features that eluted from the HILIC column and have a database match with a log *D* < 0 at pH 7

**Fig. 4:**
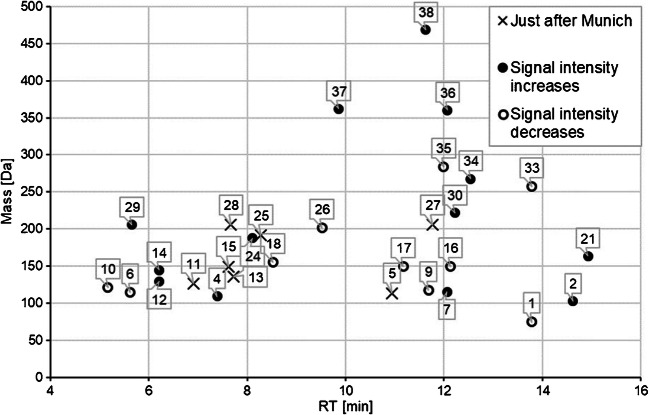
The dot plot shows features that were detected in the March samples either upstream or downstream of Munich (circles, location IDs 8 and 9, Table S1) or just downstream of Munich (crosses). The features eluted from the HILIC column and were proposed by the STOFF-IDENT database. Only matches with a negative log *D* value were considered. The numbers indicate the ID of the database queries as are listed in Table 2

The spatial and temporal distribution of these features across all 33 samples is illustrated in Fig. 3. The study indicates that the overall number of features in the Isar river was significantly higher in March than in May or July of 2015. This result could be attributed to a concentration effect due to a relatively low precipitation (26 mm) in February of the same year. In contrast, there were 80 mm and 58 mm of rainfall in April and May, respectively (measured in Garmisch by the Bavarian State Ministry for the Environment) [40]. However, in all campaigns, the number of features sharply increased downstream of the city of Munich. As an example, the dot plot in Fig. 4 shows the polar features detected in March around Munich. It indicates whether the feature’s absolute abundance increases or decreases downstream of Munich. Even though no definite quantitative statement can be made, comparing the signal intensities at different locations or dates could help in prioritizing features. For example, the peak height of the feature with the mass/RT coordinate (102.0547 Da/14.62 min) increased by a factor of 34. The compound proposed for that feature (ID 2 in Table 2) by the STOFF-IDENT database was guanylurea, which is an aerobic bacterial degradation product of the antidiabetic drug metformin [41]. Since guanylurea is stable against further photo- and biodegradation, it distributes over the entire regional water cycle, to such an extent that it has already been detected in the North Sea [42].

#### Confirmation using a reference standard

By using the feature’s signal intensity for rudimentary prioritization, we uncovered guanylurea (ID 2) as a compound of interest because its signal intensity increased significantly downstream of the city of Munich. In order to achieve a higher level of confidence in compound identification, a reference standard was measured under the same chromatographic and mass spectrometric conditions. Additionally, melamine (ID 11) and 1,3-dimethylimidazolidin-2-one (DMI, ID 6) were exemplarily confirmed by matching their RT and accurate mass matching with the respective reference standards. The respective EICs (Figs. S1–S3) and isotopic ratios (Table S8) are provided in the ESM. The information is summarized in Table 3. In each case, the RT deviation was below 5%, and the mass difference was below 5 ppm. MS/MS matching would be additionally required for a level 1 identification according to Schymanski et al. [43].

**Table 3:** Three tentative compounds found by the nontarget screening approach that were confirmed via RT and accurate mass matching with a reference standard. For each compound, the monoisotopic mass of the underlying feature is shown as well as the mass difference. The reference RT is the mean value of the reference standard’s RT measured *n* times. ΔRT describes the RT deviation of the molecular feature from the reference standard

Name	InChi key	Elemental formula	Log *D* (pH 7)	Monoisotopic mass (Da)	Mass difference (ppm)	Reference RT (min)	Δ RT (%)
Guanylurea	BKMMTJMQCTUHRP-UHFFFAOYSA-N	C2H6N4O	− 2.1	102.0542	4.9	14.4 (*n* = 5)	1.4
Melamine	JDSHMPZPIAZGSV-UHFFFAOYSA-N	C3H6N6	− 2.0	126.0654	0.8	7.0 (*n* = 5)	− 1.3
1,3-Dimethylimidazolidin-2-one	CYSGHNMQYZDMIA-UHFFFAOYSA-N	C5H10N2O	− 0.6	114.0793	1.8	5.9 (*n* = 2)	− 4.8

However, validation by means of RT and MS matching could resolve ambiguous allocations, as the example of melamine shows: For the three features A (126.0655 Da/6.9 min), B (126.0651 Da/7.2 min), and C (126.0665 Da/8.4 min), the STOFF-IDENT database output was melamine. The RT of the reference standard of 7.0 min closely matched the RT of feature A. In addition, the absolute mass deviation of 0.0001 Da was the lowest of the three.

The feature of guanylurea was detected 13 times in total at locations 2, 7, 8, 9, and 10 (ESM Table S1).

The feature allocated to melamine was found in four samples at locations 9, 10, and 11. The findings are in line with the expectation that pharmaceuticals and their transformation products primarily occur downstream of urban areas.

One newfound polar substance of emerging interest is 1,3-dimethylimidazolidin-2-one (DMI). The feature of DMI was detected in all eleven samples from March 2015 and compared with the reference standard. It is a high-boiling aprotic solvent that, according to the European Chemicals Agency (ECHA), is used in pH regulators, water treatment products, and laboratory chemicals. It is registered by six active suppliers under REACH and 100–1000 t is brought into the European Economic Area per year. The Predicted No-Effect Concentration in freshwater is 100 μg L^−1^ [44]. To the best of our knowledge, there is currently no detailed information available regarding the occurrence and distribution of DMI in the aquatic environment.

## Electronic supplementary material

ESM 1 (PDF 830 kb).
