# Correction to: Optimized hidden target screening for very polar molecules in surface waters including a compound database inquiry

**DOI:** 10.1007/s00216-021-03427-z

**Published:** 2021-06-02

**Authors:** Susanne Minkus, Sylvia Grosse, Stefan Bieber, Sofia Veloutsou, Thomas Letzel

**Affiliations:** 1 Technical University of Munich (Chair of Urban Water Systems Engineering), Am Coulombwall 3, 85748 Garching, Germany; 2 Analytisches Forschungsinstitut für Non-Target Screening GmbH (AFIN-TS), Am Mittleren Moos 48, 86167 Augsburg, Germany; 3 Present Address: Thermo Fisher Scientific, Dornierstraße 4, 82110 Germering, Germany; 4 Present Address: N. Votsi 35, 10445, Athens, Greece

**Correction to: Analytical and Bioanalytical Chemistry (2020) 412:4953–4966**

10.1007/s00216-020-02743-0

The article Optimized hidden target screening for very polar molecules in surface waters including a compound database inquiry, written by Susanne Minkus, Sylvia Grosse, Stefan Bieber, Sofia Veloutsou, Thomas Letzel, was originally published Online First without Open Access. After publication in volume 412, issue 20, page 4953–4966 the author decided to opt for Open Choice and to make the article an Open Access publication. Therefore, the copyright of the article has been changed to ©The Author(s) 2021 and the article is forthwith distributed under the terms of the Creative Commons Attribution 4.0 International License, which permits use, sharing, adaptation, distribution and reproduction in any medium or format, as long as you give appropriate credit to the original author(s) and the source, provide a link to the Creative Commons licence, and indicate if changes were made. The images or other third party material in this article are included in the article’s Creative Commons licence, unless indicated otherwise in a credit line to the material. If material is not included in the article’s Creative Commons licence and your intended use is not permitted by statutory regulation or exceeds the permitted use, you will need to obtain permission directly from the copyright holder. To view a copy of this licence, visit http://creativecommons.org/licenses/by/4.0.

