# Supplementary material for: Optimized hidden target screening for very polar molecules in surface waters including a compound database inquiry
Source: Anal Bioanal Chem. 2020 Jun 2;412(20):4953–66. doi: 10.1007/s00216-020-02743-0 (PMC8206052; doi:10.1007/s00216-020-02743-0)
Supplement: Supplementary file 1 — (PDF 830 kb). [file 216_2020_2743_MOESM1_ESM.pdf]

Analytical and Bioanalytical Chemistry

Electronic Supplementary Material

Optimized hidden target screening for very polar molecules in surface waters including a compound database inquiry

Susanne Minkus, Sylvia Grosse, Stefan Bieber, Sofia Veloutsou, Thomas Letzel

Table S1 Sample locations and coordinates. The relevance of the sampling location is explained in the comment. Each location was sampled during March, May and July of 2015. The samples 1, 7 and 10 of March and July were used for the DoE

ID	Location	Coordinates	Comment
**1**	Austria	47.395241, 11.265037	Estimation of the water quality before entering Germany
**2**	After Mittenwald	47.518692, 11.295976	Downstream of a wastewater treatment plant (WWTP) that discharges into the Isar river and a dam
**3**	Seinsbach river	47.473354, 11.279471	Pristine from the mountains
**4**	After Sylvenstein Lake	47.639632, 11.593347	Reservoir that creates long hydraulic retention times
**5**	Jachen river	47.639859, 11.576852	Carries water from a storage power plant
**6**	After Bad Tölz	47.781697, 11.545989	Sampling site is located 800 m downstream of a WWTP
**7**	Loisach river	47.933574, 11.428642	700 m downstream of a WWTP and 500 m upstream of the Loisach river entering the Isar
**8**	Before Munich	48.040292, 11.513920	Right before Isar enters the city of Munich
**9**	After Munich	48.313040, 11.699037	Located 2 km after a WWTP that treats water of Munich
**10**	After Freising	48.403349, 11.792101	2.2 km downstream of the WWTP that belongs to the town of Freising
**11**	Dingolfing	48.634222, 12.494877	Located downstream of the town of Landshut and a local power plant

Table S2 Information on the gradients for the RPLC separation and the HILIC separation

**RPLC**			**HILIC**		
Time [min]	Flow rate [mL min^{-1}]	Solvent B [%]	Time [min]	Flow rate [mL min^{-1}]	Solvent D [%]
0	0.05	0	0	0.40	0
7	0.05	0	6	0.40	0
12	0.05	50	13	0.40	40
13	0.10	50	32	0.40	40
22	0.10	100	33	0.80	0
32	0.10	100	53	0.80	0
33	0.10	0	54	0.40	0
53	0.10	0	58	0.40	0
54	0.05	0			
58	0.05	0			

Table S3 The 68 standard compounds shown below were measured four times over the course of the campaign. The mean values and the standard deviations (SDs) were calculated for the retention time (RT), the mass error (deviation of the observed mass from the monoisotopic mass), the full width at half maximum (FWHM) of the peak and its height. For the parameter RT, the relative standard deviation (RSD) is also provided. The entries are sorted in ascending order by mean RT

Compound Name	Formula	InChi Key	Monoisotopic Mass	Mean RT [min]	SD RT [min]	RSD RT [%]	Mean mass error [ppm]	SD mass error [ppm]	Mean FWHM [min]	SD FWHM [min]	Mean height [Counts]	SD height [Counts]
Dimethylsulfoxide	C2 H6 O S	IAZDPXIOMUYVGZ-UHFFFAOYSA-N	78.0139	5.9	0.1	1.1	5.0	2.1	0.24	0.04	37291	63734
Methylurea	C2 H6 N2 O	XGEGHDBEHXKFPX-UHFFFAOYSA-N	74.0480	6.0	0.1	1.4	7.8	1.3	0.20	0.03	491597	912742
2-pyrrolidinone	C4 H7 N O	HNJBEVLQSNELDL-UHFFFAOYSA-N	85.0528	6.1	0.1	1.7	3.0	1.4	0.14	0.02	105865	196125

Compound Name	Formula	InChi Key	Monoisotopic Mass	Mean RT [min]	SD RT [min]	RSD RT [%]	Mean mass error [ppm]	SD mass error [ppm]	Mean FWHM [min]	SD FWHM [min]	Mean height [Counts]	SD height [Counts]
Dicyandiamide	C2 H4 N4	QGBSISYHAICWAH-UHFFFAOYSA-N	84.0436	6.2	0.2	2.6	6.4	1.3	0.18	0.04	97936	169073
1-methylimidazole	C4 H6 N2	MCTWTZJPVLRJOU-UHFFFAOYSA-N	82.0531	6.3	0.1	1.1	5.7	3.4	0.17	0.07	4838967	8795216
6-Mercaptopurine	C5 H4 N4 S	GLVAUDGFNGKCSF-UHFFFAOYSA-N	152.0157	6.5	0.2	3.7	3.1	0.7	0.36	0.14	286785	406380
4(5)-Methylimidazole	C4 H6 N2	XLSZMDLNRCVEIJ-UHFFFAOYSA-N	82.0531	6.7	0.1	1.2	5.6	2.2	0.26	0.06	5055221	6614692
Adenine	C5 H5 N5	GFFGJBXGBJISGV-UHFFFAOYSA-N	135.0545	6.9	0.1	1.3	1.2	1.6	0.20	0.01	1037819	1839883
Melamine	C3 H6 N6	JDSHMPZPIAZGSV-UHFFFAOYSA-N	126.0654	7.0	0.1	0.8	2.2	2.4	0.34	0.06	1932888	3131122
Riboflavin	C17 H20 N4 O6	AUNGANRZJHBGPY-UHFFFAOYSA-N	376.1383	7.3	0.1	1.6	4.0	2.4	0.33	0.02	538360	848416
L-phenylalanine	C9 H11 N O2	COLNVLDHVKWLRT-QMMMGPOBSA-N	165.0790	10.3	0.2	1.5	4.4	3.8	0.25	0.17	162023	184999
Gabapentin	C9 H17 N O2	UGJMXCAKCUNAIE-UHFFFAOYSA-N	171.1259	10.4	0.5	4.6	3.4	2.5	0.29	0.20	844230	1188812
Leucine	C6 H13 N O2	ROHFNLRQFUQHCH-UHFFFAOYSA-N	131.0946	10.4	0.0	0.4	3.6	1.5	0.26	0.18	272597	371024
L-isoleucin	C6 H13 N O2	AGPKZVBTJJNPAG-WHFBIAKZSA-N	131.0946	10.8	0.1	0.7	2.2	1.7	0.20	0.12	263624	337823
L-tryptophan	C11 H12 N2 O2	QIVBCDIJIAJPQS-VIFPVBQESA-N	204.0899	10.9	0.3	2.9	3.4	0.8	0.24	0.12	72993	88316
2,2',2''-nitrilotriethanol	C6 H15 N O3	GSEJCLTVZPLZKY-UHFFFAOYSA-N	149.1067	11.4	0.2	2.0	8.8	7.1	0.31	0.30	825943	1515162
L-tyrosine	C9 H11 N O3	OUYCCCASQSFEME-QMMMGPOBSA-N	181.0739	11.6	0.1	1.0	3.1	0.3	0.23	0.07	55310	41879
Betaine	C5 H11 N O2	KWIUHFFTVRNATP-UHFFFAOYSA-N	117.0790	11.8	0.0	0.4	0.6	1.9	0.41	0.12	1238247	2295923
Atenolol	C14 H22 N2 O3	METKIMKYRPQLGS-UHFFFAOYSA-N	266.1630	12.0	0.5	3.8	3.9	2.3	0.36	0.14	2401537	3362767
Glutamate	C5 H9 N O4	WHUUTDBJXJRKMK-VKHMYHEASA-N	147.0532	12.1	0.1	0.8	2.2	1.3	0.21	0.05	111016	164053
Vigabatrin	C6 H11 N O2	PJDFLNIOAUIZSL-UHFFFAOYSA-N	129.0790	12.7	0.0	0.3	2.1	0.8	0.28	0.09	616169	887394
L-asparagine	C4 H8 N2 O3	DCXYFEDJOCDNAF-REOHCLBHSA-N	132.0535	13.1	0.1	1.0	2.6	1.9	0.44	0.20	80562	98645
Sotalol	C12 H20 N2 O3 S	ZBMZVLHSJCTVON-UHFFFAOYSA-N	272.1195	14.5	0.6	4.2	3.5	1.8	1.13	0.43	726289	926508
Guanylurea	C2 H6 N4 O	SQSPRWMERUQXNE-UHFFFAOYSA-N	102.0542	14.7	0.4	2.4	2.1	1.2	0.48	0.32	736194	1038678
Metformin	C4 H11 N5	XZWYZXLIPXDOLR-UHFFFAOYSA-N	129.1014	17.0	0.8	4.4	1.8	1.3	0.99	0.72	997888	1229991
Primidone	C12 H14 N2 O2	DQMZLTXERSFNPB-UHFFFAOYSA-N	218.1055	23.7	0.2	0.7	5.4	1.5	0.21	0.09	25964	20892
Haloxyfop	C15 H11 Cl F3 N O4	GOCUAJYOYBLQRH-UHFFFAOYSA-N	361.0329	25.5	0.3	1.1	4.3	3.8	0.20	0.05	29582	26386
Diclofenac	C14 H11 Cl2 N O2	DCOPUUMXTXDBNB-UHFFFAOYSA-N	295.0167	25.7	0.4	1.4	4.4	1.9	0.23	0.05	20882	22389
Carbetamide	C12 H16 N2 O3	AMRQXHFXNZFDCH-VIFPVBQESA-N	236.1161	25.8	0.2	0.9	5.6	1.7	0.15	0.09	1391259	2136834
Indomethacin	C19 H16 Cl N O4	CGIGDMFJXJATDK-UHFFFAOYSA-N	357.0768	25.9	0.4	1.7	5.1	1.6	0.13	0.00	109787	113961
Oxadixyl	C14 H18 N2 O4	UWVQIROCRJWDKL-UHFFFAOYSA-N	278.1267	26.3	0.3	1.0	2.2	4.8	0.14	0.04	1834928	2030737
Monuron	C9 H11 Cl N2 O	BMLIZLVNXIYGCK-UHFFFAOYSA-N	198.0560	26.3	0.2	0.9	3.9	1.0	0.16	0.01	951744	1623685
Carbamazepine	C15 H12 N2 O	FFGPTBGBLSHEPO-UHFFFAOYSA-N	236.0950	26.5	0.2	0.9	2.7	5.3	0.13	0.02	2349907	2997974
Phenytoin	C15 H12 N2 O2	CXOFVDLJLONNDW-UHFFFAOYSA-N	252.0899	26.5	0.2	0.9	5.1	1.2	0.13	0.01	75960	110426
Azamethiphos	C9 H10 Cl N2 O5 P S	VNKBTWQZTQIWDV-UHFFFAOYSA-N	323.9737	26.5	0.2	0.9	5.5	2.9	0.12	0.06	2878677	5067968
Atrazine	C8 H14 Cl N5	MXWJVTOOROXGIU-UHFFFAOYSA-N	215.0938	27.7	0.3	1.0	6.2	3.4	0.13	0.05	2477966	3226190
N,N-diethyl-m-toluamide (DEET)	C12 H17 N O	MMOXZBCLCQITDF-UHFFFAOYSA-N	191.1310	27.7	0.3	0.9	5.4	4.0	0.12	0.05	3132623	3800263
Carboxin	C12 H13 N O2 S	GYSSRZJIHXQEHQ-UHFFFAOYSA-N	235.0667	27.8	0.2	0.8	3.4	1.0	0.16	0.01	902560	970027
Testosterone	C19 H28 O2	MUMGGOZAMZWBJJ-DYKIIFRCSA-N	288.2089	28.0	0.3	1.0	3.5	3.9	0.16	0.02	115814	111034

Compound Name	Formula	InChi Key	Monoisotopic Mass	Mean RT [min]	SD RT [min]	RSD RT [%]	Mean mass error [ppm]	SD mass error [ppm]	Mean FWHM [min]	SD FWHM [min]	Mean height [Counts]	SD height [Counts]
Metobromuron	C9 H11 Br N2 O2	WLFDQEVORAMCIM-UHFFFAOYSA-N	258.0004	28.2	0.2	0.7	2.7	6.5	0.12	0.07	40185	20351
Metazachlor	C14 H16 Cl N3 O	STEPQTYSZVCJPV-UHFFFAOYSA-N	277.0982	28.3	0.2	0.8	4.7	2.9	0.17	0.03	2946569	4054204
Diphenhydramine	C17 H21 N O	ZZVUWRFHKOJYTH-UHFFFAOYSA-N	255.1623	28.8	0.6	2.0	3.9	2.3	0.29	0.14	3218370	3525887
Flurtamone	C18 H14 F3 N O2	NYRMIJKDBAQCHC-UHFFFAOYSA-N	333.0977	28.9	0.2	0.8	2.9	2.7	0.13	0.07	2163773	2057452
Tris(2-chloro-1-methylethyl) phosphate (TCPP)	C9 H18 Cl3 O4 P	KVMPUXDNESXNOH-UHFFFAOYSA-N	326.0008	29.0	0.2	0.8	4.2	2.2	0.18	0.01	303175	203903
Diethofencarb	C14 H21 N O4	LNJNFVJKDJYTEU-UHFFFAOYSA-N	267.1471	29.2	0.2	0.8	3.8	0.7	0.16	0.02	239752	149112
Linuron	C9 H10 Cl2 N2 O2	XKJMBINCVNINCA-UHFFFAOYSA-N	248.0119	29.3	0.2	0.8	2.0	1.9	0.18	0.00	73997	94418
Norfluoxetine	C16 H16 F3 N O	WIQRCHMSJFFONW-UHFFFAOYSA-N	295.1184	29.5	0.6	2.1	3.6	2.5	0.30	0.15	959864	1503514
Chlorbromuron	C9 H10 Br Cl N2 O2	NLYNUTMZTCLNOO-UHFFFAOYSA-N	291.9614	29.6	0.2	0.8	3.5	1.9	0.18	0.01	77590	103420
Boscalid	C18 H12 Cl2 N2 O	WYEMLYFITZORAB-UHFFFAOYSA-N	342.0327	29.8	0.2	0.8	3.1	1.9	0.16	0.01	141605	69289
Molinate	C9 H17 N O S	DEDOPGXGGQYYMW-UHFFFAOYSA-N	187.1031	30.2	0.2	0.8	6.0	1.1	0.15	0.03	69840	97180
Methyl dihydrojasmonate	C13 H22 O3	KVWWIYGFBYDJQC-UHFFFAOYSA-N	226.1569	30.3	0.2	0.7	4.7	6.2	0.15	0.02	31169	25586
Malathion	C10 H19 O6 P S2	JXSJBGJIGXNWCI-UHFFFAOYSA-N	330.0361	30.4	0.2	0.7	4.2	0.6	0.16	0.03	859644	684270
Fenoxycarb	C17 H19 N O4	HJUFTIJOISQSKQ-UHFFFAOYSA-N	301.1314	30.4	0.2	0.7	6.8	1.0	0.15	0.02	208143	174020
Metconazol	C17 H22 Cl N3 O	XWPZUHJBOLQNMN-UHFFFAOYSA-N	319.1451	30.5	0.2	0.7	4.4	2.8	0.14	0.05	3123646	3307415
Flufenacet	C14 H13 F4 N3 O2 S	IANUJLZYFUDJIH-UHFFFAOYSA-N	363.0665	30.6	0.2	0.7	3.7	1.3	0.16	0.01	264444	112156
Metolachlor	C15 H22 Cl N O2	WVQBLGZPHOPPFO-UHFFFAOYSA-N	283.1339	30.8	0.2	0.7	4.7	1.8	0.20	0.03	1727283	1622800
Alachlor	C14 H20 Cl N O2	XCSGPAVHZFQHGE-UHFFFAOYSA-N	269.1183	30.8	0.2	0.7	3.5	0.8	0.36	0.03	105024	69633
Tris[2-chloro-1-(chloromethyl)ethyl] phosphate (TDCPP)	C9 H15 Cl6 O4 P	ASLWPAWFJZFCKF-UHFFFAOYSA-N	427.8839	31.0	0.2	0.7	3.4	2.4	0.15	0.00	41791	17891
Chlorfenvinphos	C12 H14 Cl3 O4 P	FSAVDKDHPDSCTO-UHFFFAOYSA-N	357.9695	31.1	0.2	0.8	5.4	1.5	0.13	0.03	955520	608441
Oxybenzone	C14 H12 O3	DXGLGDHPHMLXJC-UHFFFAOYSA-N	228.0786	31.1	0.2	0.6	4.0	1.3	0.18	0.01	194328	149481
Picoxystrobin	C18 H16 F3 N O4	IBSNKSODLGJUMQ-UHFFFAOYSA-N	367.1031	31.3	0.2	0.7	2.2	1.8	0.19	0.03	3929775	2355193
Pyraclostrobin	C19 H18 Cl N3 O4	HZRSNVGNWUDEFX-UHFFFAOYSA-N	387.0986	32.1	0.2	0.7	-0.4	1.8	0.18	0.08	7563212	4124593
Triclocarban	C13 H9 Cl3 N2 O	ICUTUKXCWQYESQ-UHFFFAOYSA-N	313.9780	32.3	0.2	0.6	-0.9	1.3	0.19	0.02	44608	6709
Diazinon	C12 H21 N2 O3 P S	FHIVAFMUCKRCQO-UHFFFAOYSA-N	304.1010	32.4	0.2	0.7	3.6	2.1	0.17	0.02	6467634	7187032
Profenofos	C11 H15 Br Cl O3 P S	QYMMJNLHFKGANY-UHFFFAOYSA-N	371.9351	33.7	0.4	1.1	2.1	1.9	0.31	0.10	193587	206316
Prosulfocarb	C14 H21 N O S	NQLVQOSNDJXLKG-UHFFFAOYSA-N	251.1344	34.1	0.2	0.7	4.5	1.5	0.18	0.03	581161	684205
Quinoxyfen	C15 H8 Cl2 F N O	WRPIRSINYZBGPK-UHFFFAOYSA-N	306.9967	34.5	0.2	0.5	1.5	1.4	0.17	0.07	3678364	1956188
Fenofibrat	C20 H21 Cl O4	YMTINGFKWWXKFG-UHFFFAOYSA-N	360.1128	35.1	0.2	0.7	2.3	1.7	0.21	0.02	78572	77999

Table S4 The eight quantitative factors were defined at two levels. Those minimum and maximum values either derived from the operational range given by the program or an estimation based on the initial raw data check. The objective of a design of experiment is to check a reduced set of factor value combinations. The experimental plan displayed below derives from a Plackett-Burman screening design (experiments 1-12) with a complete fold-over (experiments 13-24), two adjusted center-point runs (experiments 25, 26) and two runs to check for non-linearities of factor (experiments 27, 28)

Exp. No.	F1 [Counts]	F2 [-]	F3 [min]	F4 [ppm]	F5 [Counts]	F6 [Counts]	F7 [Counts]	F8 [ppm]
**1**	2000	1	1.0	5.0	0	5000	5000	10.0
**2**	2000	4	0.1	50.0	0	5000	0	10.0
**3**	0	4	1.0	5.0	5000	0	0	10.0
**4**	2000	1	1.0	50.0	0	0	0	500.0
**5**	2000	4	0.1	50.0	5000	0	5000	10.0
**6**	2000	4	1.0	5.0	5000	5000	0	500.0
**7**	0	4	1.0	50.0	0	0	5000	500.0
**8**	0	1	1.0	50.0	5000	5000	5000	10.0
**9**	0	1	0.1	50.0	5000	5000	0	500.0
**10**	2000	1	0.1	5.0	5000	0	5000	500.0
**11**	0	4	0.1	5.0	0	5000	5000	500.0
**12**	0	1	0.1	5.0	0	0	0	10.0
**13**	0	4	0.1	50.0	5000	0	0	500.0
**14**	0	1	1.0	5.0	5000	0	5000	500.0
**15**	2000	1	0.1	50.0	0	5000	5000	500.0
**16**	0	4	0.1	5.0	5000	5000	5000	10.0
**17**	0	1	1.0	5.0	0	5000	0	500.0
**18**	0	1	0.1	50.0	0	0	5000	10.0
**19**	2000	1	0.1	5.0	5000	5000	0	10.0
**20**	2000	4	0.1	5.0	0	0	0	500.0
**21**	2000	4	1.0	5.0	0	0	5000	10.0
**22**	0	4	1.0	50.0	0	5000	0	10.0
**23**	2000	1	1.0	50.0	5000	0	0	10.0
**24**	2000	4	1.0	50.0	5000	5000	5000	500.0
**25**	1000	2	0.6	27.5	2500	2500	2500	200.0
**26**	1000	2	0.6	27.5	2500	2500	2500	200.0
**27**	1000	3	0.1	27.5	2500	2500	2500	100.0
**28**	1000	3	1.0	27.5	2500	2500	2500	100.0

Table S5 Below the mean values and standard deviations (SD) of the ten features are given which responses R5 and R6 are based on. The integration of all their EICs was manually checked based on the following criteria: The relative mass differences of the peaks within a compound group should not exceed an overall spread of ± 10 ppm. Furthermore, a peak is approximately Gaussian shaped and in its mass spectrum, the monoisotopic signal should be accompanied by at least one additional isotope ion

Feature	Mean RT [min]	SD RT [min]	Mean mass	SD mass
**1**	13.2	0.0	75.0326	0.0003
**2**	7.2	0.0	117.0789	0.0001
**3**	6.3	0.1	144.0906	0.0005
**4**	27.7	0.1	227.1898	0.0008
**5**	5.8	0.0	268.1523	0.0007
**6**	9.3	0.0	314.1945	0.0008
**7**	29.9	0.3	414.2073	0.0016
**8**	26.0	0.1	529.3828	0.0013
**9**	23.7	0.1	848.4306	0.0062
**10**	11.9	0.0	921.0027	0.0001

Table S6 The responses were calculated after conducting the 28 experiments given in Table S4. Each set of six response values corresponds to the result of an experiment. At the bottom the specifications that were defined for the limit optimization are provided

Exp. No.	R1 [min]	R2 [%]	R3 [%]	R4 [ppm]	R5 [-]	R6 [-]
**1**	0.07	2.6	7.9	1.5	6	15
**2**	0.03	2.7	16.0	1.5	4	25
**3**	0.33	5.8	44.8	2.6	5	15
**4**	0.22	5.6	35.8	2.3	6	20
**5**	0.03	1.7	8.4	1.2	4	22
**6**	0.22	7.0	35.4	2.7	9	19
**7**	0.13	2.7	24.4	2.4	6	13
**8**	0.07	2.4	9.6	1.6	4	10
**9**	0.02	3.3	11.4	1.8	5	24
**10**	0.02	3.4	5.2	1.6	2	27
**11**	0.02	3.2	12.7	1.8	4	17
**12**	0.03	2.7	13.1	1.6	2	16
**13**	0.03	3.3	14.5	1.9	5	19
**14**	0.11	4.2	18.6	2.2	6	17
**15**	0.02	3.0	5.6	1.5	2	26
**16**	0.03	1.9	10.0	1.4	3	21
**17**	0.26	6.0	38.8	2.6	12	13
**18**	0.03	1.8	6.7	1.3	3	23
**19**	0.03	2.6	9.8	1.4	4	27
**20**	0.03	3.5	13.7	1.8	4	23
**21**	0.09	2.5	12.1	1.6	6	13
**22**	0.32	5.6	46.8	2.6	10	6
**23**	0.29	5.7	45.3	2.1	9	10
**24**	0.11	4.1	21.3	2.0	6	17
**25**	0.07	3.6	21.2	2.1	10	14
**26**	0.07	3.6	21.2	2.1	10	14
**27**	0.03	3.1	14.2	1.8	4	18
**28**	0.13	4.6	22.7	2.4	6	17
**Target**	0.01	1.0	10.0	1.0	3	7
**Maximum**	0.30	6.0	50.0	3.0	15	20

Table S7 For each response variable (R1 – 6) the coefficients and the standard errors of the model terms that are included in the model are presented. The R^2 and Q^2 values measure the goodness-of-fit and goodness-of prediction for each response. The formulas are presented at the bottom of the table with the observed value y, the predicted value ŷ, the mean value $\overline{y}$ and the predicted response $\hat{y}_{i/i}$ when leaving out the i-th object from the training set (cross-validation*). A model with a value of 1 for both, R^2 and Q^2, fits the data perfectly

	**R1**	**R2**	**R3**	**R4**	**R5**	**R6**
**Constant**	-1.185 ± 0.024	3.673 ± 0.135	1.351 ± 0.079	2.136 ± 0.123	10.000 ± 1.023	14.300 2.132
**F1**	-	-	-0.043 ± 0.023	-0.110 0.035	-	2.083 ± 0.614
**F2**	-	-	0.076 ± 0.023	0.091 ± 0.035	-	-
**F3**	0.385 ± 0.025	0.868 ± 0.140	0.186 ± 0.022	0.310 ± 0.034	1.731 ± 0.284	-3.962 ± 0.590
**$F3^2$**	-	-	-0.157 ± 0.082	-0.240 ± 0.127	-4.731 ± 1.062	3.959 ± 2.210
**F4**	-	-	-	-	-	-
**F5**	-	-	-	-	-	-
**F6**	-	-		-	0.458 ± 0.295	-
**F7**	-0.120 ± 0.026	-0.851 ± 0.145	-0.170 ± 0.023	-0.195 ± 0.035	-0.958 ± 0.295	-
**F8**	-	0.441 ± 0.143	-	0.161 ± 0.035	-	1.329 ± 0.605
$\boldsymbol{R^2 = 1 - \frac{\sum_{i=1}^{n}(y_i-\hat{y}_i)^2}{\sum_{i=1}^{n}(y_i-\bar{y}_i)^2}; Q^2 = 1 - \frac{\sum_{i=1}^{n}(y_i-\hat{y}_{i/i})^2}{\sum_{i=1}^{n}(y_i-\bar{y}_i)^2}}$						
**R^2**	0.913	0.775	0.868	0.880	0.753	0.739
**Q^2**	0.889	0.685	0.796	0.805	0.651	0.638

* Golub GH, Heath M, Wahba G (1979) Generalized cross-validation as a method for choosing a good ridge parameter. Technometrics 21:215–223.

Table S8 The table shows isotope ratios of the chromatographic peaks from the EICs displayed in the Figures S1-3. The highest mass spectrometric peak was scaled to 100%. When calculating isotope ratios mass spectrometric peaks with intensities < 1% were neglected. Deviations of one percentage point could occur for low-intensity peaks of molecules with more than five atoms of the same kind*

	**Peak**	**Intensity (expected) [%]**	**Intensity (reference) [%]**	**Intensity (samples) [%]**
**Guanylurea**	First isotope	100.0	100.0	100.0
	Second isotope	3.7	3.1	3.0
**Melamine**	First isotope	100.0	100.0	100.0
	Second isotope	5.5	4.6	4.4
**1,3-dimethylimidazolidin-2-one**	First isotope	100.0	100.0	100.0
	Second isotope	6.3	5.8	3.3

* Pfeilsticker D Isotopen-Muster-Rechner. https://www.pfeilsticker.net/chemie/massen/. Accessed 25 Jan 2020

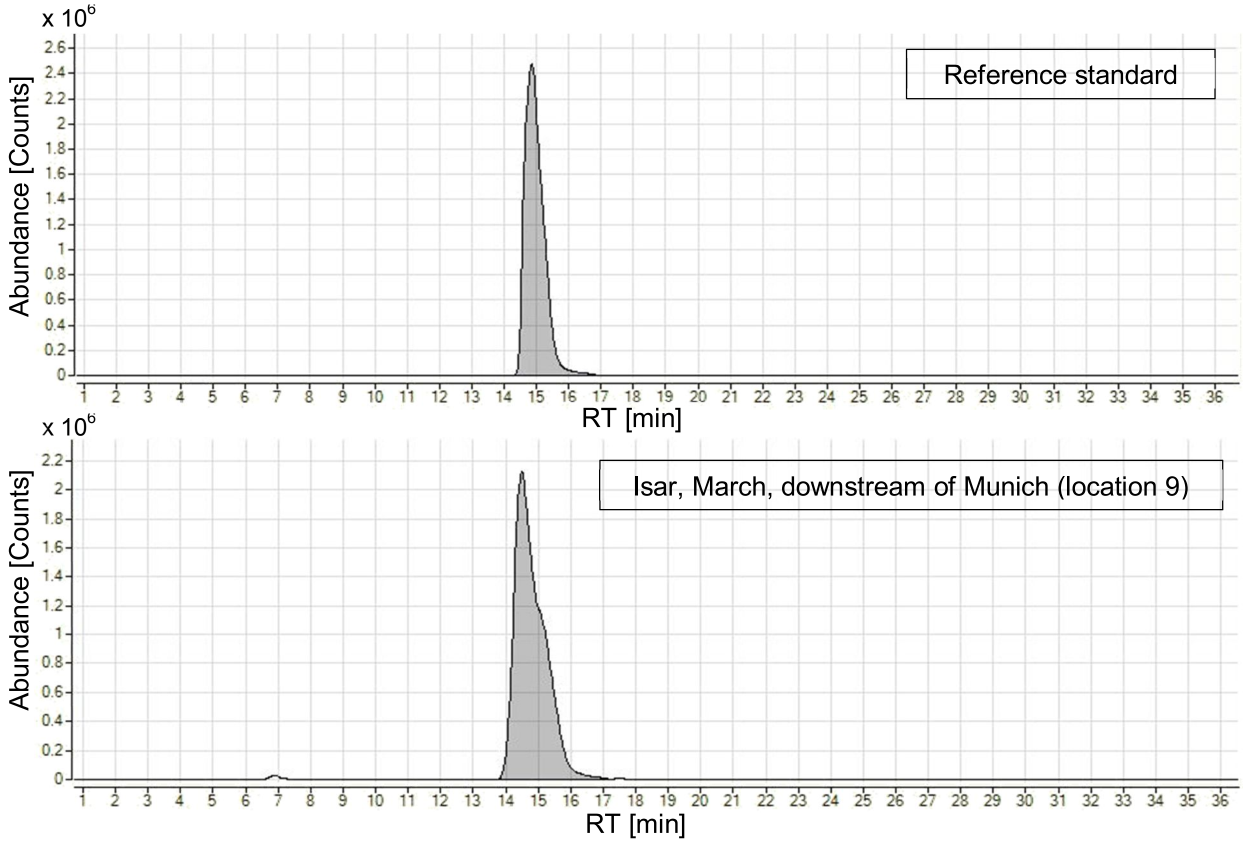

Fig. S1 Extracted ion chromatograms (EICs) of guanlyurea (ID 2) in the standard mix (top) and in the water sample (bottom)

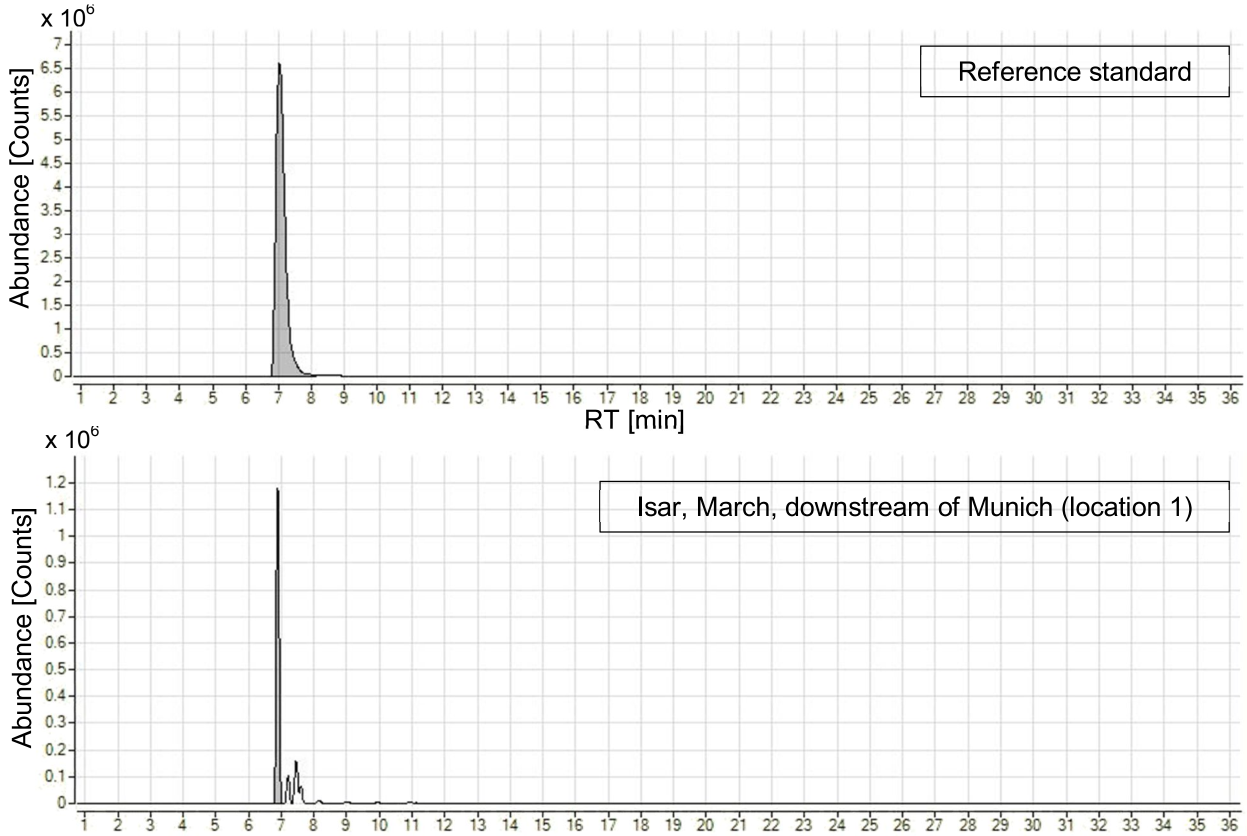

Fig. S1 Extracted ion chromatograms (EICs) of melamine (ID 11) in the standard mix (top) and in the water sample (bottom)

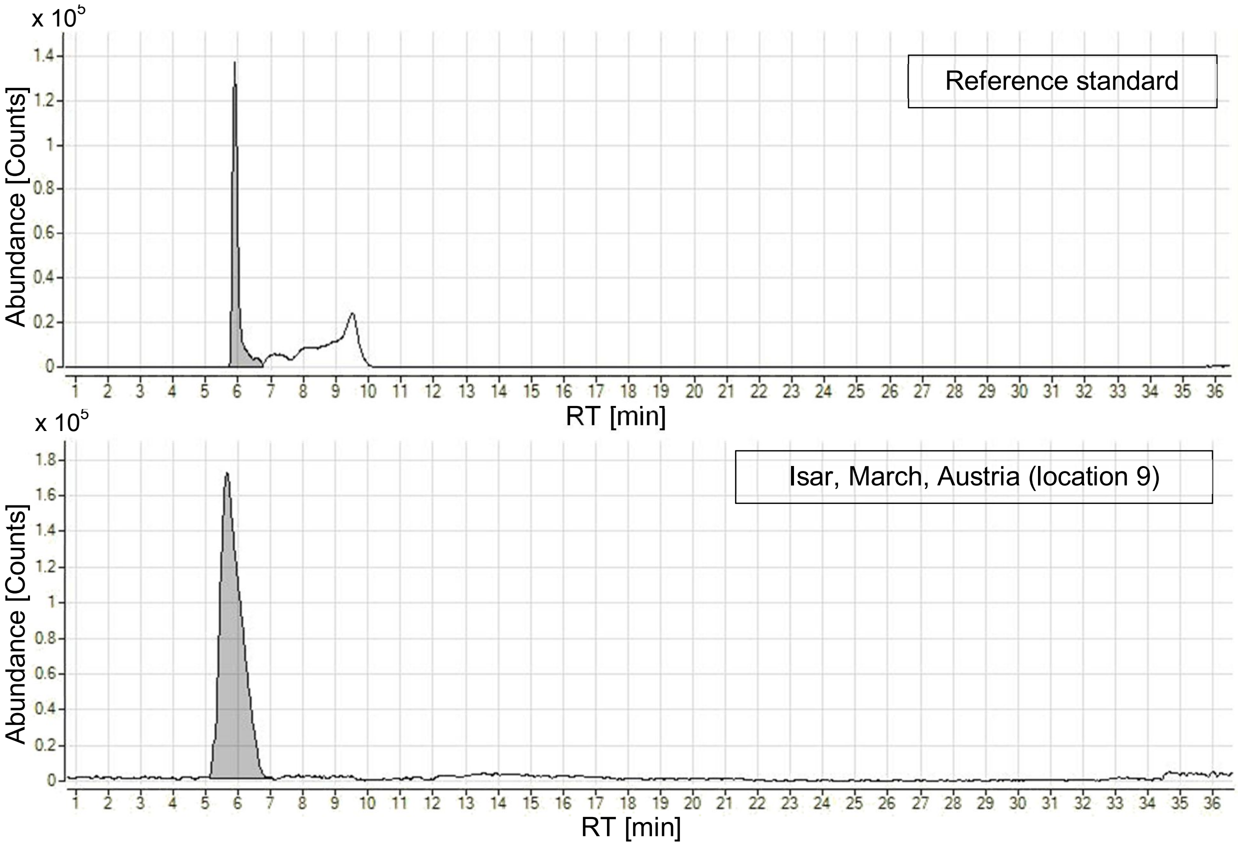

Fig. S2 Extracted ion chromatograms (EICs) of 1,3-dimethylimidazolidin-2-one (ID 6) in the standard mix (top) and in the water sample (bottom)
